# Chinese medical named entity recognition utilizing entity association and gate context awareness

**DOI:** 10.1371/journal.pone.0319056

**Published:** 2025-02-25

**Authors:** Yang Yan, Yufeng Kang, Wenbo Huang, Xudong Cai

**Affiliations:** Institution of Computer Science and Technology, Changchun Normal University, Changchun, Jilin, China; The University of Lahore, PAKISTAN

## Abstract

Recognizing medical named entities is a crucial aspect of applying deep learning in the medical domain. Automated methods for identifying specific entities from medical literature or other texts can enhance the efficiency and accuracy of information processing, elevate medical service quality, and aid clinical decision-making. Nonetheless, current methods exhibit limitations in contextual awareness and insufficient consideration of contextual relevance and interactions between entities. In this study, we initially encode medical text inputs using the Chinese pre-trained RoBERTa-wwm-ext model to extract comprehensive contextual features and semantic information. Subsequently, we employ recurrent neural networks in conjunction with the multi-head attention mechanism as the primary gating structure for parallel processing and capturing inter-entity dependencies. Finally, we leverage conditional random fields in combination with the cross-entropy loss function to enhance entity recognition accuracy and ensure label sequence consistency. Extensive experiments conducted on datasets including MCSCSet and CMeEE demonstrate that the proposed model attains F1 scores of 91.90% and 64.36% on the respective datasets, outperforming other related models. These findings confirm the efficacy of our method for recognizing named entities in Chinese medical texts.

## 1 Introduction

Named Entity Recognition (NER) is a fundamental task within the field of Natural Language Processing (NLP), encompassing the automated identification of entities with specific meanings, including individuals’ names, locations, organizations, and more, within textual data [1]. This task plays a pivotal role across various domains, including but not limited to information retrieval, knowledge graph construction, question answering systems, content recommendation, sentiment analysis, and automatic summarization. Within the medical domain, named entity recognition (NER) holds significant importance. NER in medicine finds extensive applications in clinical decision support, public health monitoring, data standardization, and personalized medicine integration by automatically identifying medically relevant entities, including diseases, symptoms, drug names, medical procedures, and patient information, from sources like medical literature, clinical records, and trial reports [2].

Currently, in medical named entity recognition research, statistical and lexicon-based methods as well as machine learning-based methods have historically made contributions, but deep learning-based approaches have emerged as the prevailing trend. This trend can be attributed to the capability of deep learning models (e.g., LSTM [3], GRU [4], and Transformer [5]) to automatically extract intricate features from raw text, adeptly leverage contextual cues, handle unstructured data accurately, and exhibit strong generalization capabilities. Nevertheless, current methods exhibit limitations in contextual awareness and tend to overlook the contextual relationships and interactions between entities, which can be primarily characterized by:

1) Limited contextual understanding: While deep learning models excel at capturing local contextual information, they still face challenges in comprehending broader contextual environments and long-range dependencies, particularly within complex medical contexts.
2) Inadequate recognition of entity associations: Current deep learning models may not adequately recognize and leverage complex relationships among medical entities, such as those between diseases and corresponding treatments, which are vital for enhancing the accuracy and clinical applicability of named entity recognition.

Addressing these limitations, we introduce a method for Chinese medical named entity recognition method utilizing recurrent neural attention networks. This method leverages the RoBERTa-wwm-ext pre-trained model designed to capture comprehensive contextual information and integrates recurrent neural networks with an attention mechanism to comprehensively understand and represent medical entities and their interrelations.Specifically, the main innovations and contributions of this study are summarized as follows:

1) Innovative application of recurrent neural attention networks: We introduce a novel recurrent neural attention network model designed specifically for named entity recognition in Chinese medical texts. This model leverages the dynamic routing capability of recurrent neural networks to capture intricate entity relationships. Additionally, it integrates a multi-head attention mechanism to augment the model’s capacity for focusing on crucial information, offering a fresh perspective on Chinese medical named entity recognition.
2) Multi-level feature extraction and fusion: We devised a multi-level feature extraction framework capable of extracting rich features across three levels: character, word, and sentence levels. By integrating recurrent neural networks with attention mechanisms, the model can more accurately comprehend and represent medical entities along with their contextual relationships.
3) Optimized utilization of gating mechanism: We introduce an innovative gating mechanism into the model to dynamically adjust the fusion degree of features across various levels. This mechanism enables the model to flexibly manage entity features based on contextual cues, thereby improving the model’s adaptability and accuracy in processing complex medical texts.
4) Experimental validation and performance enhancement: We conduct extensive experiments on two benchmark datasets for Chinese medical named entity recognition to validate the effectiveness of the proposed model. The experimental results demonstrate significant improvements in both entity recognition precision and recall compared to existing mainstream methods, particularly in dealing with complex and nested entities.

In summary, this study not only introduces an innovative entity recognition model for Chinese medical naming but also empirically validates its efficacy in enhancing recognition performance and managing intricate entity relationships. These findings offer novel insights and groundwork for further exploration and optimization of Chinese medical text processing techniques.

## 2 Related work

This section is dedicated to exploring related work in the realm of Chinese medical named entity recognition. These approaches can be classified into two primary research directions based on their emergence order and escalating significance: methods based on machine learning and deep learning.

Early named entity recognition (NER) methods in the medical field primarily relied on traditional machine learning techniques. Certain researchers employed algorithms like Support Vector Machine (SVM) and Conditional Random Fields (CRF) for clinical entity recognition. Saifullah et al. [6] devised a support vector machine-based approach that targets named entities in Chinese bioinformatics text summaries. Zhang et al. [7] explored Conditional Random Fields-based named entity recognition in Chinese medical records, extracting dictionary features via a medical dictionary-based approach. Dash et al. [8] organized multiple features of the support vector machine using a bag-of-words model. Zhen et al. [9] performed research experiments on word segmentation and entity recognition employing a dual decomposition model. These studies showcase a variety of methods and techniques in Chinese medical text processing. While they excel in specific skin cancer classification tasks, they often only identify a subset of named entities and lack generalization across a broader spectrum of entity types due to limited feature selection.

A significant recent advancement in deep learning-based medical named entity research is the integration of the ATTENTION mechanism into neural networks for named entity recognition. In particular, Iqbal et al. [10] employed neural networks and multilayer convolutional neural networks to analyze extensive medical texts. hmed et al. [11] utilized BiLSTM as the foundational Named Entity Recognition (NER) structure, whereas Arslan et al. [12] integrated BiLSTM with CRFs to enhance drug name recognition efficiency. Building on this, Li et al. [13] introduced a BiLSTM-Att-CRF-based clinical named entity recognition method for Chinese EHRs, a model adept at capturing extensive contextual information and mitigating long-distance information loss, thereby particularly enhancing the recall rate. Additionally, Waghela et al. [14] employed an enhanced BERT architecture pretrained on unlabeled Chinese medical text to enhance the accuracy of clinical named entity recognition. Uparkar et al. [15] extracted insights and strategies from the Transformer model to enhance the accuracy of convolutional neural networks, whereas Cevik et al. [16] introduced a pre-trained medical sequence labeling model for the task, which integrates pooled context embedding. These studies collectively illustrate the promising application of deep learning in processing complex medical texts and enhancing the accuracy and efficiency of clinical data processing tasks. However, the aforementioned methods still exhibit deficiencies in contextual understanding and entity relevance recognition. Present research methodologies fail to fully account for the intricate interactions among medical entities and multi-level semantic dependencies, essential for precise recognition and comprehension of medical texts. Addressing the aforementioned limitations, this paper introduces a novel method for Chinese medical named entity recognition using recurrent neural attention networks. The model, built upon the RoBERTa-wwm-ext [17] pre-trained model, captures abundant contextual information and incorporates recurrent neural network architectures along with a multi-head attention mechanism to comprehensively comprehend and represent medical entities and their interrelations.

## 3 Method

**Fig 1:**
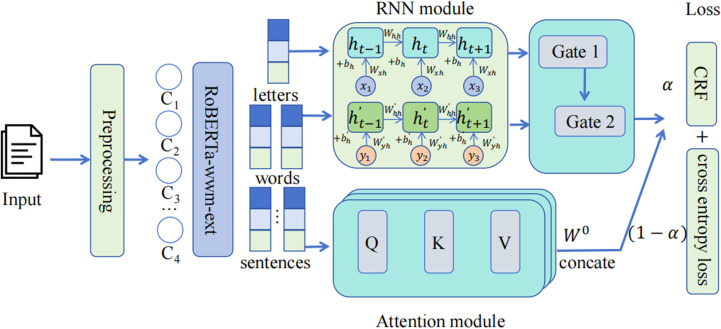
Framework overview for our suggested model.

To improve the model’s contextual awareness and its ability to focus on the relevance between entities and their interactions, we propose a method that employs a recurrent neural network along with a dynamic gating mechanism, which is builds upon a pre-trained model, functioning as the primary feature extraction layer. The overall architecture is illustrated in Fig 1:

### 3.1 Chinese medical text encoder

Since the focus of this paper is on named entities within the medical domain, which entail more specialized and complex terminology and expressions compared to general-purpose domains, choosing a suitable pre-trained model as the encoder is crucial for improving the accuracy of entity recognition. In this study, we employ the RoBERTa-wwm-ext pre-trained model, which is built on the Transformer architecture, as an encoder to more effectively capture the intricate semantic and terminological characteristics present in medical texts.

RoBERTa-wwm-ext’s core is built upon the Transformer architecture, comprising multiple layers of self-attention mechanisms. Each layer comprises a Multi-Head Attention mechanism and a fully connected feed-forward network. This design enables the model to simultaneously attend to multiple positions in a sentence, thereby more effectively capturing long-distance dependencies. Compared to the original BERT model, RoBERTa-wwm-ext demands a longer training time, a larger batch size, and more training data. These optimizations contribute to the model achieving higher accuracy in understanding semantics. The architecture of RoBERTa-wwm-ext is well-suited for medical named entity recognition tasks due to the complex terminology and long-distance semantic dependencies often found in medical texts. The self-attention mechanism of RoBERTa-wwm-ext can effectively capture these features. For instance, when analyzing a text detailing pathology features, the model can comprehend the relationships between different medical terms, even when they are widely dispersed within the sentence.

To enhance the performance of RoBERTa-wwm-ext on medical texts, we conducted the following adaptation steps: firstly, we subjected RoBERTa-wwm-ext to additional pre-training on extensive corpora from the medical domain. These corpora encompass various sub-domains ranging from pharmacology and pathology to clinical medicine, enabling the model to gain a deeper understanding of the linguistic features and terminology specific to the medical domain. To better align with the characteristics of medical texts, we expanded the model’s vocabulary by incorporating additional medical terminology. This expansion aims to minimize occurrences of out-of-vocabulary terms and enhance the model’s accuracy in processing specialized texts. Additionally, we fine-tuned the model’s attention mechanism to prioritize medical entities and their contextual information. This enhancement enables the model to accurately identify and classify medical named entities, including diseases, medications, and medical procedures.

To improve the model’s effectiveness in medical named entity recognition tasks, this paper employs RoBERTa-wwm-ext and BiLSTM as the core processing units. This integrated approach aims to harness the robust contextual understanding of RoBERTa-wwm-ext and the sequence information capturing capabilities of BiLSTM to accurately recognize and classify entities in medical texts. We impose a flexible length limit on the Chinese character count of the input text. Considering the complexity and information density of medical texts, we opt for a relatively lengthy limit, specifically setting the maximum input text length to 512 Chinese characters. This length constraint is intended to balance the model’s computational efficiency with its ability to capture long-range dependencies, ensuring adequate contextual information capture. Additionally, to better align with the characteristics of medical texts, we introduce specific preprocessing strategies for the input text. Taking the example text [“眼”, “睛”, “痛”, “肿”, “胀”, “是”, “什”, “么”, “原”, “因”], we label these terms with entities using the BIO tagging scheme, where “B” signifies the beginning of an entity, “I” represents the continuation of an entity, “E” indicates the end of an entity, and “O” stands for non-entity. In the given example, we denote two entities, “眼睛痛” and “肿胀”, with the label sequence [B, I, E, B, E, O, O, O, O, O, O]. In this context, the primary function of the BiLSTM layer is to capture contextual information in both directions within a text sequence. For an input sequence $X=\left(x_{1},x_{2},\ldots ,x_{n}\right)$BiLSTM handles the information according to the following equation:


$$\begin{array}{c} \vec{h_{t}}=LSTM\left(\vec{h_{t-1},}x_{t}\right),\overset{\leftarrow }{h_{t}}=LSTM\left(\overset{\leftarrow }{h_{t+1}},x_{t}\right)\\ h_{t}=\left[\vec{h_{t}};\overset{\leftarrow }{h_{t}}\right] \end{array}$$ (1)

Where ${\text{h}}_{\text{t}}$ represents the forward LSTM. During each time step t in the sequence, the forward LSTM receives the input ${\text{x}}_{\text{t}}$ from the current time step along with the hidden state from the preceding step $\vec{{\text{h}}_{\text{t}-1}}$ of the previous time step, computing the hidden state $\vec{{\text{h}}_{\text{t}-1}}$ of the present time step. $\overset{\leftarrow }{{\text{h}}_{\text{t}}}$ represents the reverse LSTM, which operates similarly but in the reverse direction. At each time step t, the reverse LSTM handles the input ${\text{x}}_{\text{t}}$ of the current time step and the hidden state of the subsequent time step $\overset{\leftarrow }{{\text{h}}_{\text{t}+1}}$ to produce the hidden state for the present time step $\overset{\leftarrow }{{\text{h}}_{\text{t}}}$Subsequently, the hidden states of the forward and reverse LSTMs are merged to produce ${\text{h}}_{\text{t}}$representing the final hidden state for the current time step. This linkage guarantees that the hidden state at each time step incorporates data from both directions of the sequence. In the realm of medical named entity recognition, this configuration allows the BiLSTM to take into account contextual information surrounding medical terms, which is essential for accurately interpreting their meanings and delineating entity boundaries. For instance, a specific medical term may have diverse meanings in different contexts, and BiLSTM can better capture this complexity due to its bidirectional nature.

### 3.2 Chinese medical named entity encoder

The medical named entity encoder introduced in this paper utilizes a multi-level architecture that integrates recurrent neural networks (RNNs) and multi-head attention mechanisms. This design effectively handles Chinese medical text data at multiple hierarchical levels, enhancing the accuracy of recognizing and comprehending medical named entities and their complex interrelations.

#### 3.2.1 Character-level sequence processing.

To identify linguistic features at the character level in medical texts, particularly focusing on special characters or abbreviations in medical terminology, we incorporate Recurrent Neural Networks (RNNs) for character-level sequences. RNNs efficiently process both character-level and phrase-level sequence data by iteratively handling each element in the sequence and preserving a hidden state to capture accumulated information. This aids in the model’s comprehension of the contextual and linguistic structure within the text. For a character sequence $X=\left(x_{1},x_{2},\ldots ,x_{n}\right)$, its formal representation is as follows:


$$h_{t}=f\left(W_{hh}h_{t-1}+W_{xh}x_{t}+b_{h}\right)$$ (2)

Here, $x_{t}$ signifies the character input at time step *t*, while $h_{t}$ represents the associated hidden state. The weight matrices are denoted as $W_{hh}$ and $W_{xh}$, $b_{h}$ is the bias term, and *f* refers to the activation function. The RNN layer enables the model to understand language patterns at the character level by sequentially processing each character and updating the hidden state, thus retaining information from the sequence up to that moment.

#### 3.2.2 Phrase-level sequence processing.

To capture phrase-level semantic and syntactic structures in medical texts, we employ the same RNN structure for processing phrase-level sequences. The processing of phrase-level RNNs resembles that of character-level ones. For a phrase sequence $Y=\left(y_{1},y_{2},\ldots ,y_{n}\right)$, the formula is given as follows:


$$h_{t}^{'}=f\left(W_{hh}^{'}h_{t-1}^{'}+W_{yh}^{'}y_{t}+b_{h}^{'}\right)$$ (3)

Here, $y_{t}$ represents the phrase input at time step t and $h_{t}^{\text{'}}$ denotes the corresponding hidden state. This layer assists the model in comprehending the relationships between phrases and their roles in the overall semantic structure.

#### 3.2.3 Sentence-level complex relationship processing.

The multi-head attention mechanism is utilized to analyze intricate relationships at the sentence level. In medical texts, relationships between entities often span multiple dimensions, encompassing various levels of semantic and contextual information. By concurrently processing this information, the multi-head attention framework enables the model to integrate the significance of entities within a sentence and their interrelationships from diverse perspectives. Multi-head attention facilitates the model to simultaneously attend to different facets of the sentence. Particularly in medical texts, this architecture enables the model to comprehensively capture intricate interactions between entities, such as the correlation between symptoms and diseases, or the interplay between drugs and diseases. Additionally, each “head” in the multi-head attention mechanism concentrates on a distinct segment of the sequence, offering a more holistic understanding of the entity and its surrounding context. This is essential for discerning the diverse meanings of medical terms across various contexts. Here, the fundamental equation of the multi-head attention mechanism is provided:


$$MultiHead\left(Q,K,V\right)=Concat(head_{1},head_{2},\ldots ,head_{m})W^{O}$$ (4)

$$head_{i}=Attention\left(QW_{i}^{Q},KW_{i}^{K},VW_{i}^{V}\right)$$ (5)

$$Attention\left(Q,K,V\right)=softmax(\frac{QK^{T}}{\sqrt{d_{k}}})V$$ (6)

Here, *Q*, *K*, and *V* denote queries, keys, and values, which may either be the output ℎ′ or other suitable representations derived from the phrase-level RNN. The parameters $W_{i}^{Q}，W_{i}^{K}，W_{i}^{V}$, and $W^{O}$ represent the learnable weight matrices within the model. The term $d_{k}$ represents the size of the keys, utilized for scaling the dot product. The variable *m* refers to the number of attention heads, allowing the model to simultaneously capture different pieces of information. This setup allows the submodule designed for handling complex relationships at the sentence level to efficiently detect and examine intricate connections between entities in medical texts, thereby improving the precision of named entity recognition and relationship extraction.

### 3.3 Gate control information flow processing unit

To improve the effectiveness of the Chinese medical named entity encoder, we incorporate two gating units into the model. These gating units regulate the flow and integration of character-level, phrase-level, and sentence-level information to ensure that the model can effectively fuse and utilize information from different levels. For the character-level and phrase-level gating units, we position them between the character-level RNN and the phrase-level RNN. Their role is to determine the amount of information flow between character-level and phrase-level information. These gating units help the model determine which level of information is more important in a particular context, thus improving the accuracy of entity recognition. Its formula is expressed as follows:


$$\begin{array}{c} g_{t}=\sigma \left(W_{g}\left[h_{t};h_{t}^{'}\right]b_{g}\right)\\ {\tilde{h}}_{t}^{'}=g_{t}⊙h_{t}^{'}+\left(1-g_{t}\right)⊙h_{t} \end{array}$$ (7)

Here, $h_{t}$ represents the output of the character-level RNN, $h_{t}^{\text{'}}$ represents the output of the phrase-level RNN, $g_{t}$ represents the output of the gating unit, $W_{g}$ represents the weight matrix, $b_{g}$ represents the bias term, *σ* represents the sigmoid activation function, and  ⊙  denotes element-by-element multiplication.

Similarly, we position the gating unit between the phrase-level processing module and the sentence-level multi-head attention mechanism to regulate the combination of phrase-level and sentence-level information. This ensures that the model analyzes complex relationships at the sentence level while fully considering phrase-level details. Its formula is expressed as follows:


$$\begin{array}{c} g_{t}^{'}=\sigma \left(W_{g}^{'}\left[{\tilde{h}}_{t}^{'};H_{t}\right]+b_{g}^{'}\right)\\ {\tilde{H}}_{t}=g_{t}^{'}⊙H_{t}+\left(1-g_{t}^{'}\right)⊙{\tilde{h}}_{t}^{'} \end{array}$$ (8)

Here, ${\tilde{h}}_{t}^{\text{'}}$ represents the phrase-level RNN output adjusted by the first gating unit, ${\tilde{H}}_{t}$ represents the output of sentence-level multi-head attention, $g_{t}^{\text{'}}$ represents the output of the gating unit, $W_{g}^{\text{'}}$ represents the weight matrix, and $b_{g}^{\text{'}}$ represents the bias term. Introducing gating units between different levels enables the model to process multilevel information in medical texts more flexibly and effectively. This method strengthens the model’s grasp of the boundaries and internal composition of medical entities, as well as its ability to detect complex medical terminology and the interrelations between entities.

### 3.4 Loss function

To improve the effectiveness of the medical named entity recognition task, choosing an appropriate loss function is crucial. In medical text named entity recognition, we need to focus not only on the correct classification of individual labels but also on the overall correctness of the label sequence. Therefore, in this paper, we use a composite loss function that combines cross-entropy loss and conditional random field loss.

The cross-entropy loss evaluates the prediction accuracy of the model for each individual label. For the medical named entity recognition task, it can be defined as follows:


$$L_{CE}=-\overset{n}{\underset{t=1}{\sum }}\overset{C}{\underset{c=1}{\sum }}y_{t,c}log{\hat{y}}_{t,c}$$ (9)

Here, *n* represents the sequence length, *C* represents the number of labeled categories, $y_{t,c}$ represents the true label (0 or 1) of time step *t* for category *c*, and ${\hat{y}}_{t,c}$ represents the corresponding predicted probability of the model.

Based on this rationale, we introduce the CRF loss to consider the overall correctness of label sequences, particularly the dependencies between labels. The loss function of the CRF layer can be expressed as follows:


$$L_{CRF}=-logP\left(y|x\right)$$ (10)

where $P\left(y|x\right)$ denotes the conditional probability of the labeled sequence *y* given the input sequence *x*, as calculated by the CRF layer based on the model.

Combining the above components, our composite loss function is as follows:


$$L=\alpha L_{CE}+\left(1-\alpha \right)L_{CRF}$$ (11)

Here, *α* is an adjustable parameter that balances the weight of cross-entropy loss and CRF loss in the total loss. Adjusting the weight parameter α allows for finding an optimal balance between precise identification of individual entities and the overall accuracy of the label sequence, resulting in higher recognition performance.

## 4 Experiment and result

### 4.1 Experimental data set

In this research, we employ the publicly accessible Chinese medical text datasets MCSCSet [18] and CMeEE [19] to assess the performance of our named entity recognition model. These datasets comprise texts from medical papers, clinical reports, and online medical consultations, encompassing a broad spectrum of medical entity types including dis-eases, symptoms, and drugs. Each dataset underwent rigorous preprocessing and an-notation to ensure the accuracy and reliability of the experiments. In both datasets, MCSCSet is an extensively annotated dataset specifically designed for Chinese spelling correction within the medical field. Unlike current open-domain datasets for Chinese spelling correction, MCSCSet incorporates a large number of real-world med-ical queries gathered from Tencent Medical Dictionary, along with corresponding misspelled sentences manually annotated by medical experts. Table 1 displays the statistics for the MCSCSet dataset:

**Table 1:** Shows the distribution statistics of entities in the MCSCSet dataset.

Sample size	Training sets	Validators	Test sets	Medical entities
196,496	137,547	29,449	29,449	81,020

CMeEE (Chinese Medical Named Entity Recognition Dataset) is tailored for recognizing named entities in the medical field within Chinese text. The dataset was originally released in CHIP2020, where the task involves identifying and extracting entities from a given sentence based on a predefined pattern and categorizing them into nine classes: diseases, clinical manifestations, drugs, medical devices, medical procedures, body parts, medical examinations, microorganisms, and departments, etc. Table 2 displays the statistics for the CMeEE dataset:

**Table 2:** Shows the distribution statistics of entities in the CMeEE dataset.

Sample size	Training sets	Validators	Test sets
23,000	15,000	5,000	3,000

For data preprocessing, we iterate through each sentence in both datasets, segmenting it into individual characters and labeling them based on whether they belong to an entity or not. We utilize “B”, “I”, “E”, and “O” to indicate the beginning, inside, end, and non-entity parts of an entity, respectively. Corresponding target labels are generated for each character accordingly.

### 4.2 Parameterisation and evaluation criteria

The experimental setup in this study consists of an Intel(R) Xeon(R) CPU operating at 2.40 GHz and an NVIDIA GeForce RTX 3090 GPU with 24 GB of VRAM. The software environment uses Python version 3.9 and PyTorch version 1.13. The hyperparameters of the model are set to 100 epochs, batch size is 16, learning rate is 5 × 10^ − 5^, learning rate decay is 0.01, maximum text length is 512, and BiLSTM dimension is 512. To validate the effectiveness of the model, we select precision (*P*), recall (*R*), and F1 score as the evaluation metrics, with their corresponding formulas presented as follows:


$$P=\frac{TP}{TP+FP}$$ (12)

$$R=\frac{TP}{TP+FN}$$ (13)

$$F1=2\times \frac{P\times R}{P+R}$$ (14)

Where TP and FP represent the number of entities identified correctly and the number of entities identified incorrectly, and FN is the number of entities that identify positive examples as negative examples.

### 4.3 Experimental setup

This paper conducts a sufficiently large number of experiments on 2 datasets, and to guarantee the validity of the findings, we employ the cross-validation method. Specifi-cally, we partition each dataset into five subsets, utilizing four for training the model and another for validation. This process is repeated five times for each dataset, selecting a different subset for validation each time while using the other subsets for training. This method ensures that the model undergoes evaluation on the complete dataset, thus enhancing the consistency and reliability of the outcomes. To evaluate the effectiveness of our proposed model, we will conduct experiments in conjunction with the following pre-trained models, as detailed below:

BERT-base [20]: BERT-base is one of the original BERT models developed by the Google AI team. It employs a bi-directional Transformer architecture and is pre-trained on large textual datasets to acquire profound linguistic representations. The BERT-base version comprises 12 Transformer layers, relatively smaller in size.

BERT-wwm [21]: BERT-wwm enhances BERT by employing a Whole Word Mask-ing (WWN) strategy, where during pre-training, not only individual words but entire words are masked. This strategy is especially effective for languages like Chinese.

MacBERT-base [22]: MacBERT, an advancement over the BERT model, particu-larly focuses on the masking strategy. It employs a technique known as “Masked Lan-guage Model (MLM) masking” to enhance pre-training.

Ernie-1 [23]: Ernie-1, a pre-training model developed by Baidu, incorporates sup-port for knowledge graphs and enhances the processing of entity- and phrase-level in-formation compared to BERT.

RoBERTa [24]: RoBERTa, an optimized version of BERT developed by Facebook AI, enhances performance by eliminating the downstream task pre-training objectives of BERT and fine-tuning the hyperparameters. RoBERTa adopts a whole-word masking strategy, ideal for processing Chinese corpus.

RoBERTa-wwm-ext [25]: RoBERTa-wwm-ext, an extension of RoBERTa-wwm, typically undergoes pre-training on larger datasets for improved performance. It in-herits the optimizations of RoBERTa and further enhances the model by pre-training on a broader range of data.

In this paper, we assess the performance of the proposed gated context-aware model for named entity recognition by comparing it with the aforementioned methods in experiments conducted on medical datasets MCSCSet and CMeEE. The experimental findings are displayed in Table 3 and Table 4. In the MCSCSet dataset, under identical parameter settings, our proposed model achieves precision, recall, and F1 score of 92.36%, 91.45%, and 91.90%, respectively, outperforming all other model comparisons, demonstrating the superior performance of our proposed RoB-ERTa-wwm-ext-GCA-crf.

**Table 3:** Shows the experimental outcomes for the MCSCSet dataset.

Data set	Model	Precision(%)	Recall(%)	F1(%)
MCSCSet	BERT-base	90.22	87.41	88.79
	BERT-wwm	90.82	88.20	89.49
	MacBERT-base	89.13	87.98	88.55
	RoBERTa	92.10	90.52	91.30
	RoBERTa-wwm-ext-GCA-crf (Ours)	**92.36**	**91.45**	**91.90**

**Table 4:** Shows the experimental outcomes for the CMeEE dataset.

Data set	Model	Precision(%)	Recall(%)	F1(%)
CMeEE	MacBERT-large [26]	–	–	62.40
	BERT-Biaffine [27]	64.17	61.29	62.29
	BERT-CRF [27]	58.34	64.08	61.07
	Lattice-LSTM+Med-BERT [27]	56.84	47.58	51.80
	RoBERTa-wwm-ext-GCA-crf (Ours)	**66.43**	**63.07**	**64.36**

Based on the experimental results, RoBERTa-wwm-ext-GCA-crf demonstrates strong performance on both datasets, achieving F1 scores of 64.36 and 91.90, respectively. These scores are notably higher compared to other models including BERT-base-crf, BERT-wwm-crf, MacBERT-base-crf, and RoBERTa-wwm-crf. This outcome underscores the significant advantage of our model in handling medical texts, especially regarding recognition accuracy and recall. This advantage can be attributed to the innovative model structure, which combines RNN and multi-head attention mechanisms, along with the incorporation of gating units. The multi-head attention mechanism enables RoBERTa-wwm-ext-GCA-crf to grasp the significance of each entity and the intricate interrelations among entities within a broader context. This is essential for managing the complex, multi-faceted relationships and intricate semantic structures found in medical literature. Moreover, the gating unit effectively governs the flow of information across character, phrase, and sentence levels, ensuring efficient integration and utilization of information. These results above illustrate the efficacy and sophistication of our model in medical named entity recognition tasks.

**Fig 2:**
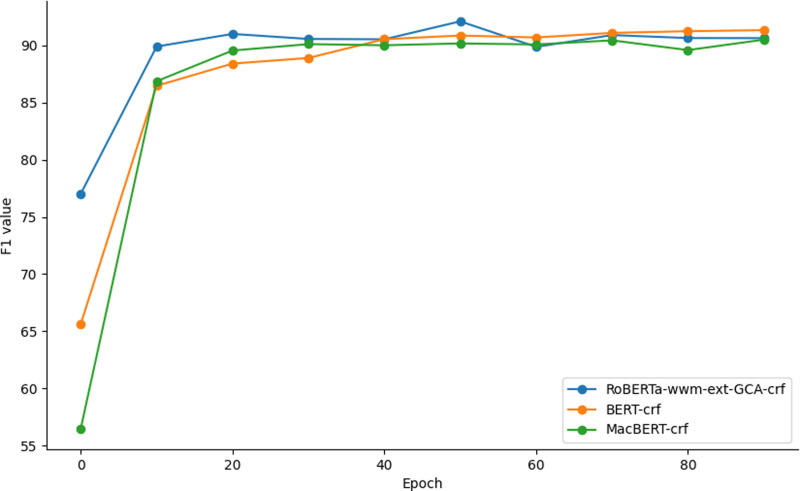
Comparison of convergence speed of different models.

Meanwhile, to investigate the convergence speed of RoBERTa-wwm-ext-GCA-crf, we conduct experiments using MCSCSet as a case study, comparing its convergence speed with that of BERT-crf, MacBERT-base-crf, and other BERT pre-trained models. The experimental results are depicted in Fig 2:

Observing the model performance during the training process, we notice that the RoBERTa-wwm-ext-GCA-crf model exhibits strong learning ability from the outset. In the initial phase, its F1 value rapidly increases to 77.01%, surpassing the initial F1 values of the BERT-crf and MacBERT-base-crf models, which stand at 65.63% and 56.48%, respectively. As training progresses, the RoBERTa-wwm-ext-GCA-crf model stabilizes after reaching its peak value. By the sixth round, its F1 value peaks at 92.08%, whereas the F1 values of the BERT-crf and MacBERT-base-crf models continue to steadily increase, reaching 90.84% and 90.15%, respectively.

It is noteworthy that the performance of the RoBERTa-wwm-ext-GCA-crf model does not show a significant decline upon reaching its peak. This may be attributed to the gated context-aware mechanism, which aids in mitigating overfitting to some extent, thus ensuring the ongoing stability of the model’s performance.

### 4.4 Ablation experiments

To ascertain whether the improved performance of the RoBERTa-wwm-ext-GCA-crf method for entity recognition is mainly attributable to the gating context-awareness feature we proposed, we carried out the following ablation experiments:

1) Exclude the gated context-aware module, retaining solely the RoBERTa-wwm-ext model along with the CRF layer, to assess the performance variation between the model lacking GCA and the complete version.
2) Maintain the gating unit while turning off the multi-head attention mechanism to evaluate how multi-head attention influences the model’s overall performance.

**Fig 3:**
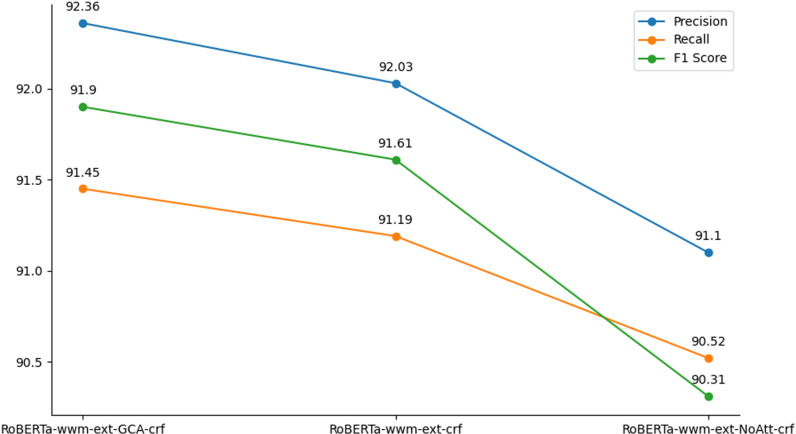
Comparison of ablation experiment results.

The experimental results of the ablation experiments are illustrated in Fig 3, where our proposed RoBERTa-wwm-ext-GCA-crf exhibits a slight performance improvement over the ablation experimental model RoBERTa-wwm-ext-crf and another benchmark model RoBERTa-crf across all three metrics.

Additionally, as depicted in Fig 3, the model demonstrates robust performance in terms of precision rate even after removing the multi-attention mechanism, closely aligning with the ablation experimental model RoBERTa-wwm-ext-crf (92.03%) with a precision rate of 91.10%, slightly below that of the original model RoBERTa-wwm-ext-GCA-crf (92.36%). However, in terms of recall, it performs slightly worse at 90.52%, lower than both the original model (91.45%) and the ablation experimental model (91.19%). This discrepancy may suggest that the model’s entity detection ability diminishes in the absence of the multi-attention mechanism. Regarding F1 values, RoBERTa-wwm-ext-NoAttn-crf achieves 91.31%, slightly below both the original model (91.90%) and the ablation experimental model (91.61%). This suggests that although RoBERTa-wwm-ext-NoAttn-crf performs well in terms of overall performance, it may not be as good as the model that includes the full attention mechanism when dealing with complex specific tasks, especially in entity detection, where the multiple attention mechanism plays an important role.

## 5 Discussion

This study proposed an innovative model RoBERTa-wwm-ext-GCA-crf for the task of named entity recognition in Chinese medicine. The model significantly improved the performance of named entity recognition in medical texts by combining the gated context-aware (GCA) mechanism, multi-head attention, and conditional random field (CRF). Experimental results show that the model outperforms existing methods on both MCSCSet and CMeEE datasets, which verifies the effectiveness of the model in processing complex medical texts. The model uses RoBERTa-wwm-ext to extract contextual features, models dependencies between entities through the GCA mechanism, and captures semantic details through multi-head attention, which significantly improves the F1 value and recall rate. However, this study also has some limitations. In terms of versatility, although the model shows significant advantages in Chinese medical texts, its adaptability to other languages and fields has not been fully verified. Future work can expand the application scope of the model through transfer learning and fine-tuning techniques, and combine cross-language pre-training models to further improve the model’s multilingual processing capabilities. In terms of complexity and scalability, the multi-head attention and BiLSTM layers of the model architecture improve performance, but also increase computational complexity, limiting the applicability of the model in large-scale data sets and real-time applications. Future optimization directions include exploring lightweight architectures and hardware acceleration technologies to achieve efficient operation of the model. The distribution analysis of attention weights can also further reveal the decision-making basis of the model and increase its credibility in the medical field. In resource-constrained scenarios, the optimization of model performance remains a focus of future research. The results of this study mainly rely on large-scale annotated data sets. In the future, prompt learning and contrastive learning strategies will be combined to improve the adaptability of the model under resource-scarce conditions.

Exploring the combination of knowledge graphs can reduce dependence on data and further enhance the generalization ability and practical value of the model. This study provides strong technical support for medical text processing by proposing an innovative Chinese medical named entity recognition model. This study not only provides a technical basis for future medical text analysis, but also provides a new perspective for the research direction of cross-domain named entity recognition.

## 6 Conclusions

This paper presents a model, RoBERTa-wwm-ext-GCA-crf, designed and implemented for the task of Chinese medical named entity recognition. he model’s core feature is the integration of the gated context awareness (GCA) mechanism, effectively merging the RoBERTa-wwm-ext pre-training model with the bidirectional long short-term memory network (BiLSTM) to enhance comprehension and processing of complex contexts and terminology in medical texts. The multi-head attention mechanism enables the model to capture subtle associations among medical entities and enhance the in-depth understanding of entity contexts. Additionally, ablation experiments were conducted to assess each component’s contribution to the model performance, particularly the significance of the multi-head attention mechanism in the overall structure. Experimental results demonstrate that the RoBERTa-wwm-ext-GCA-crf model proposed in this paper outperforms other comparative models and the ablation experimental model in terms of precision, recall, and F1 value. This confirms the effectiveness of the gated context-aware mechanism, especially in improving the recall rate and the F1 value of the combined performance metric. In addition, the ablation experiments further emphasise the key role of the multi-head attention mechanism in enhancing the model to capture complex relationships and improve the overall performance. Future work will aim to further enhance model performance by introducing cue learning and comparison learning strategies in a sample-sparse learning environment. It is anticipated that these approaches will preserve the model’s ability to efficiently recognize medical named entities despite resource constraints. Overall, this study represents significant progress in the field of Chinese medical named entity recognition, laying a solid foundation for future in-depth research and applications in related fields.
